# Supplementary material for: Cell type boundaries organize plant development
Source: eLife. 2017 Sep 12;6:e27421. doi: 10.7554/eLife.27421 (PMC5617630; doi:10.7554/eLife.27421)
Supplement: Figure 8—source data 1. — A table listing the fold change (FC) values for each biological replicate of WOX, PRS and IAA19, the mean fold change for each gene, the standard deviation values and p-values. [file elife-27421-fig8-data1.docx]

**Source data file for Figure 8**

|  |  | PRS | | WOX1 | | IAA20 |
| --- | --- | --- | --- | --- | --- | --- |
| Rep1 FC |  | 1.678900384 | | 1.478510137 | | 2.679542279 |
| Rep2 FC |  | 3.216883524 | | 1.991086008 | | 3.602300305 |
| Rep3 FC |  | 1.735102699 | | 3.763611201 | | 2.629479589 |
| Rep4 FC |  | 3.016883524 | | 1.665693641 | | 2.923774058 |
| Rep5 FC |  | 1.335102699 | | 3.099714601 | | 2.479542279 |
| Mean |  | 2.196574566 | | 2.399723118 | | 2.862927702 |
| SD |  | 0.856885899 | | 0.988002492 | | 0.44313845 |
| p-value |  | 0.0142 | | 0.0132 | | 0.0001 |
| Median |  | 1.735102699 | | 1.991086008 | | 2.679542279 |
| SEM |  | 0.383211024 | | 0.441848147 | | 0.198177539 |
| N |  | 5 | | 5 | | 5 |
| 95% confidence intervals |  | |  | |  | |
| Upper limit |  | 2.947668173 | | 3.265745486 | | 3.251355679 |
| Lower limit |  | 1.445480959 | | 1.53370075 | | 2.474499725 |
