# Supplementary material for: Cell type boundaries organize plant development
Source: eLife. 2017 Sep 12;6:e27421. doi: 10.7554/eLife.27421 (PMC5617630; doi:10.7554/eLife.27421)
Supplement: Supplementary file 1. — (A) List of primers used for generating transgenic constructs and quantitative real-time PCR. (B) Frequencies of phenotypes amongst different transgenic plant lines described in the study. For ATML1 >>REVr-2×VENUS (n = 20, number of imaged specimens) and UBQ10 >>STTM 165/166 (n = 12) transgenic plants, we used T3 generation plants for imaging that exhibited the reported phenotypes at a frequencies ranging from 70% to 90%. For ATML1 >>KAN1 GFP transgenic plants, we imaged a particular T2 line that exhibited meristem arrest after induction at a frequency of approximately 98% (n = 10). For CLV3 >>K2G transgenic lines, among 12 different T2 lines that showed leaf morphology changes and meristem arrest, we used a particular line that produced more than two leaves after meristem arrest at a frequency of 96.8% (n = 65). For ATML1 >>PHVr transgenic lines, we imaged a line that exhibited arrested organogenesis at a frequency of 33% (n = 12). An absence of phenotype was generally associated with low levels of induced transgene expression. (C) List of parameter values used in simulations. We have used the values from Heisler and Jönsson (2006), which are based on experimental estimates where applicable [file elife-27421-supp1.docx]

**Supplemental File 1A**

Primers for cloning and Q-PCR

| **Gene** | **Primer name** | **Sequence** |
| --- | --- | --- |
| *KAN1 (At5g16560)* | KAN1g F | ACGCGTTGTTTGGATGTATGACATTAAGTAAGCTAT |
| *KAN1 (At5g16560)* | KAN1g R | GGATCCGCTTTCTCGTGCCAATCTGGTCTGCCTAA |
| *KAN1 (At5g16560)* | K1cDNA F | AGATCTAACAATGTCTATGGAAGGTGTTTTCCTAGAGAAAAC |
| *KAN1 (At5g16560)* | K1 cDNA R | GGATCCGCTTTCTCGTGCCAATCTGGTCTGCCTAA |
| *CLV3 (At2g27250)* | CLV3p F | GGAATTCCGGATTATCCATAATAAAAAC |
| *CLV3 (At2g27250)* | CLV3p R | CTGCAGGTTTTAGAGAGAAAGTGACTGAGTGA |
| *CLV3 (At2g27250)* | CLV3utr F | AAACCTGCAGGGATCCGCGGC |
| *CLV3 (At2g27250)* | CLV3p R | ATAGAATATCACTAGTTAATTATCATTGGTTTAAAGTTATAG |
| *WOX1 (At3g18010)* | WOX1p F | ggtaccTCAAAACCGGTTTTTATACGACAAGAC- |
| *WOX1 (At3g18010)* | WOX1p R | ggatccTTTGGTGTGTACTTAATTTATATGTATG |
| *WOX1 (At3g18010)* | WOX1g F | gcggcagcaagatctATGTGGACGATGGGTTACAACGAAG |
| *WOX1 (At3g18010)* | WOX1g R | atagaatatcactagtACGTCACTGATGATATACTACG |
| *PRS (At2g28610)* | PRSg F | agatctGCGTACGTGTGTACGTGAATGAAAT |
| *PRS (At2g28610)* | PRSg R | ggatccAGTTTGGTACTGTCTTGTTTGGAGT |
| *PRS (At2g28610)* | PRS FP (Q-PCR) | CAACTCCAAACAAGACAGTACCA |
| *PRS (At2g28610)* | PRS RP (Q-PCR) | ACATGAATGAAACACCTGCAGA |
| *WOX1 (At3g18010)* | WOX1 FP (Q-PCR) | GCCTCCTTCGTTGTAACCCA |
| *WOX1 (At3g18010)* | WOX1 RP (Q-PCR) | GCTGTCTCTCTCCCTTCTCC |
| *IAA20 (At2g46990)* | IAA20 FP (Q-PCR) | ATGTGCAATGAGAAGAGTCACG |
| *IAA20 (At2g46990)* | IAA20 RP (Q-PCR) | TCACAGTAGACAAGAACATCTCC |
| *ACT2 (At3g18780)* | ACT2 FP (Q-PCR) | CCTGTTCTTCTTACCGAGGC |
| *ACT2 (At3g18780)* | ACT2 RP (Q-PCR) | AATTTCCCGCTCTGCTGTTG |

**Supplemental File 1B**

Frequencies of phenotypes amongst transgenic plant lines

| **Transgenic plants** | **No of T2 lines** | **T2 line phenotypes** |
| --- | --- | --- |
| *ATML1>>REVr-2×VENUS* | 31 | 15 arrest or delay of organogenesis  8 partially or completely dorsalized leaves only  8 no phenotype |
| *UBQ10>>STTM 165/166* | 26 | 6 arrested organogenesis  20 partially or completely dorsalized leaves |
| *ATML1>>PHVr* | 50 | 4 arrest or delay of organogenesis  31 partially or completely dorsalized leaves  15 no phenotype |
| *ATML1>>KAN1-GFP* | 17 | 11 meristem arrest  6 partially or fully radialized organs only |
| *CLV3>>KAN1-2×GFP* | 32 | 10 mild change in organ position  10 meristem arrest only  12 leaf morphology change and meristem arrest |

**Supplemental File 1C**

List of parameter values used in simulations.

| **Symbol** | **Value** | **Description** |
| --- | --- | --- |
| *cA* | 0.001 | Auxin production |
| *dA* | 0.001 | Auxin degradation |
| *T* | 1.3 | Active transport of auxin (PIN1-dependent) |
| *D* | 0.002 | Passive transport of auxin |
| *cP* | 0.001 | PIN1 production |
| *dP* | 0.001 | PIN1 degradation |
| *VX* | 10.0 | Maximal production rate of polarising signal X |
| *KXA* | 10.0 | Hill constant for auxin activating X |
| *nXA* | 1 | Hill coefficient for auxin activating X |
| *KXR, KXK* | 0.1 | Hill constants for REV/KAN repressing X |
| *nXR, nXR* | 2 | Hill coefficients for REV/KAN repressing X |
| *dX* | 1.0 | Degradation of polarising signal X |
| *kp* | 0.9 | Relation of symmetric vs polarized PIN1 |
| *fp* | 0.3 | Ratio between PIN1 endo/exocytosis |
